# Structural Insights into the UbiD Protein Family from the Crystal Structure of PA0254 from *Pseudomonas aeruginosa*

**DOI:** 10.1371/journal.pone.0063161

**Published:** 2013-05-09

**Authors:** Agata Jacewicz, Atsushi Izumi, Katharina Brunner, Robert Schnell, Gunter Schneider

**Affiliations:** Department of Medical Biochemistry and Biophysics, Karolinska Institutet, Stockholm, Sweden; University of Oulu, Finland

## Abstract

The 3-polyprenyl-4-hydroxybenzoate decarboxylase (UbiD) catalyzes the conversion of 3-polyprenyl-4-hydroxybenzoate to 2-polyprenylphenol in the biosynthesis of ubiquinone. *Pseudomonas aeruginosa* contains two genes (PA0254 and PA5237) that are related in sequence to putative UbiD enzymes. A bioinformatics analysis suggests that the UbiD sequence family can be divided into two subclasses, with PA5237 and PA0254 belonging to different branches of this family. The three-dimensional structure of PA0254 has been determined using single wavelength anomalous diffraction and molecular replacement in two different crystal forms to resolutions of 1.95 and 2.3 Å, respectively. The subunit of PA0254 consists of three domains, an N-terminal α/β domain, a split β-barrel with a similar fold of a family of flavin reductases and a C-terminal α/β domain with a topology characteristic for the UbiD protein family. The middle domain contains a metal binding site adjacent to a large open cleft that may represent the active site. The two protein ligands binding a magnesium ion, His188 and Glu229, invariant in the PA0254 subclass, are also conserved in a corresponding metal site found in one of the FMN binding proteins from the split β-barrel fold family. PA0254 forms, in contrast to the hexameric UbiD from *E. coli* and *P. aeruginosa*, a homo-dimer. Insertion of four residues in a loop region in the PA0254 type enzymes results in structural differences that are incompatible with hexamer assembly.

## Introduction

Ubiquinone (Coenzyme Q) functions as an electron carrier in the respiratory chains located in the plasma membrane of prokaryotes and inner mitochondrial membrane of eukaryotes. Bacterial mutants with a disrupted ubiquinone biosynthesis display a variety of pleiotropic phenotypes, for instance inability to grow on non-fermenting substrates such as maltose and succinate, while retaining the ability to utilize glucose. Furthermore, immotility under aerobic conditions and normal motility in the absence of oxygen has been observed [1]. *Ubi* mutants also show sensitivity to chlorate, thiols and oxidative stress, implicating an important and complex role of ubiquinone in cellular metabolism and survival.

**Figure 1:**
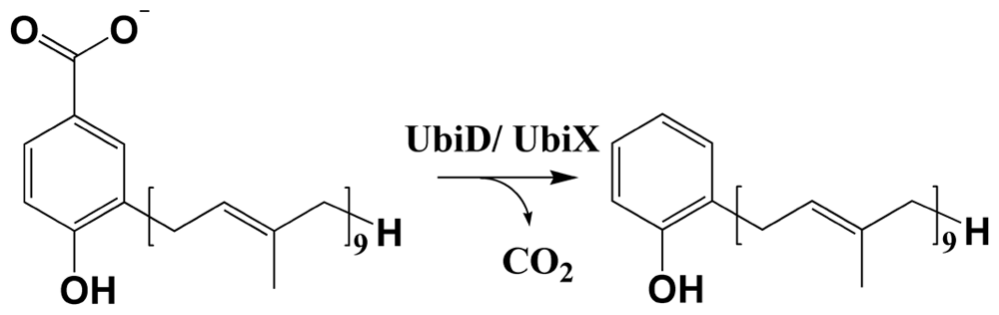
Reaction catalyzed by the putative 3-polyprenyl decarboxylases UbiD and UbiX, the decarboxylation of 3-polyprenyl-4-hydroxybenzoate to 2-polyprenylphenol. In *P. aeruginosa* ubiquinone, the polyprenyl chain consists of nine isoprene units [2].

Ubiquinone is composed of a benzoquinone ring and a hydrophobic polyprenyl chain of varying length [2]. Microorganisms synthesize ubiquinone in a similar, but not identical, manner [3], [4]. In prokaryotes, the pathway starts with the elimination of pyruvate from chorismate, the first committed step in ubiquinone biosynthesis. The resulting 4-hydroxybenzoate is prenylated at the carbon-3 position. In eukaryotes, the isoprene units are derived from the mevalonate pathway, whereas in bacteria they are synthesized via the 2-C-methylerythritol 4-phosphate (MEP) route [4]. The length of the prenyl side chain, while constant for each species, varies between organisms from 6–10 isoprene units, with typically nine units in *P. aeruginosa* [2]. After prenylation, a decarboxylation reaction leads to formation of 2-polyprenylphenol [5] (figure 1) which in a series of hydroxylation, decarboxylation and methylation reactions is converted to ubiquinone. Based on the results of genetic knock-out studies in *E. coli*, two genes, denoted *ubiX* and *ubiD*, have been assigned to encode decarboxylases that catalyze the conversion of 3-octaprenyl-4-hydroxybenzoate to 2-octaprenylphenol in bacteria and yeast [6]. UbiX and UbiD share no sequence similarity with each other and have been proposed to function as redundant enzymes. Alternatively, it has been hypothesized that they may act together in the decarboxylation step of ubiquinone synthesis.

In the human pathogen *Pseudomonas aeruginosa* PAO1, three genes (PA0254, PA4019 and PA5237) encode proteins that exhibit homology to *ubiD* or *ubiX* involved in the decarboxylation of the metabolite 3-polyprenyl-4-hydroxybenzoate in *E. coli*. PA4019 encodes a FMN-containing enzyme that is related in amino acid sequence and three-dimensional structure [7] to *E. coli* UbiX [8]. The other two proteins, PA0254 and PA5237 show sequence identities of 24% and 76%, respectively, to the putative UbiD from *E. coli*.

Random transposon mutagenesis screens of the genomes of several human pathogens and single-gene deletion studies provided evidence for the essentiality of the *ubiX* gene in *P. aeruginosa* [9], *ubiA, ubiB, ubiD* and *ubiX* in *E. coli* [10], *ubiD* and *ubiE* in *Salmonella typhimurium* [11] and *ubiA* and *ubiE* in *Helicobacter pylori* [12] thus raising the possibility that enzymes of the ubiquinone biosynthesis pathway might be potential drug targets in pathogenic bacteria. As part of a multidisciplinary program supporting early stage antimicrobial drug discovery against *P. aeruginosa* (http://www.aeropath.eu) structural studies of enzymes implicated in the ubiquinone pathway in this organism are carried out [7], [13]. Here, we report the crystal structure of the UbiD-like protein PA0254 of *P. aeruginosa* in two different crystal forms at 1.95 and 2.3 Å resolution, respectively. The enzyme subunit consists of three domains. In contrast to hexameric UbiD from *E. coli* (PDB code 2IDB) and PA5237 from *P. aeruginosa*, PA0254 forms a dimer in the crystal, and most likely also in solution. In spite of the absence of any significant overall sequence identity the fold of one of the PA0254 domains is related to a larger family of flavin binding proteins.

## Materials and Methods

### Cloning, Expression and Purification of PA0254

The full-length PA0254 gene was obtained from Genscript (Piscataway, NJ, USA) and was cloned between *Nco*I and *Hind*III sites of the pET-based pNIC28Bsa4 vector (GenBank Accession No. EF198106). The resulting plasmid expressed the PA0254 gene as a fusion protein with a TEV-cleavable N-terminal His_6_ tag and was used to transform *E. coli* BL21(DE3) competent cells. Cells containing this expression plasmid were subsequently co-transformed with the pGro7 plasmid encoding GroES and GroEL chaperones [14] for soluble protein production.

A single transformant colony was used to grow an overnight culture at 310 K. The inoculum was diluted 100-fold into 6 liters of Luria-Bertani medium containing kanamycin (35 μg/ml) and chloramphenicol (30 μg/ml) and the cells were grown at 310 K until the OD_600_ reached ∼ 0.3. Chaperone expression was induced by 2 mg/ml L-arabinose followed by the addition of IPTG to a concentration of 0.1 mM when the OD_600_ reached ∼ 0.5. At that point the temperature was decreased to 293 K and the culture was grown under inductive conditions for 18 hours. The cells were harvested by centrifugation (5000 *g*, 10 min., 277 K) and the pellets were resuspended in buffer A (50 mM Tris-HCl, pH 8.0, 100 mM NaCl, 10 mM imidazole) supplemented with 0.04 mg/ml lysozyme and 0.004 mg/ml DNAse I. The cells were lysed by sonication and the cell debris was removed by centrifugation (18000 *g*, 277 K). The clear supernatant was mixed with 1 ml Ni-NTA agarose beads (Qiagen) previously equilibrated with buffer A and incubated with gentle shaking for 30 min. at 277 K. The resin was poured into a gravity-flow column and washed with buffer A containing 15 mM imidazole. PA0254 was eluted using a non-linear 25–500 mM imidazole gradient in buffer A. Due to incomplete removal of the His_6_ tag by TEV protease in initial trials, PA0254 was subjected to further purification steps as a (His)_6_-tagged fusion. The eluted protein was loaded on the HiPrep Sephacryl S200 size-exclusion column (GE Healthcare) in buffer B (20 mM Tris-HCl, pH 7.0, 100 mM NaCl, 3 mM DTT) and concentrated to 33 mg/ml. The sample was flash-frozen in liquid nitrogen and kept at 193 K.

Selenomethionine (SeMet)-substituted PA0254 was produced in minimal M9 medium by the inhibition of methionine synthesis as described elsewhere [15] and purified using the same protocol as described above. Mass spectrometry analysis revealed a molecular mass for the SeMet-substituted protein of 57614 Da, which is very close to the calculated mass of 57616 Da for the fully substituted SeMet derivative of PA0254.

### Crystallization and Data Collection

Wild-type PA0254 crystals grew in sitting drops containing reservoir buffer (1 M (NH_4_)_2_SO_4_, 0.1 M Bis-Tris, pH 5.5, 1% PEG 3350) and protein solution (33 mg/ml in buffer B) mixed in 1∶1 volume ratio. The crystals were flash-frozen directly and a dataset to 2.30 Å resolution was collected at 100 K at beamline BM14, ESRF, Grenoble, France.

A second crystal form of wild-type PA0254 was obtained at 296 K by mixing of 2 μl of the reservoir solution (0.1 M Hepes pH 7.5, 0.2 M MgCl_2_, 10% PEG 400) with 1 μl of concentrated (30 mg/ml) protein sample and allowed to equilibrate against 0.5 ml of the well solution. The largest crystals (0.5–0.7 mm) grew within 3–5 days and were transferred to the cryo-protectant (22% (w/v) glycerol, 0.1 M Hepes 7.5, 0.2 M MgCl_2_, 3 mM DTT, 10–15% (v/v) PEG 400) and flash frozen in liquid nitrogen. A native data set from these crystals was collected at beam line ID14∶4, ESRF. Crystals of the SeMet-substituted protein were obtained under the same conditions and a SAD data set from these crystals was collected at the selenium edge to 2.7 Å resolution at 100 K at beamline I911-3, MAX IV- Laboratory, Lund, Sweden.

The X-ray data of both SeMet-substituted and wild-type PA0254 were processed with XDS [16] and SCALA of the CCP4 suite [17]. Native PA0254 crystallized in space group *P*2_1_ (a = 104.3 Å, b = 55.8 Å, c = 198.4 Å, β = 90.9°) in the presence of (NH_4_)_2_SO_4_ as precipitant, whereas crystallization using PEG 400 resulted in space group *P*3_2_21 with cell dimensions a = b = 96.8 and c = 301.7 Å (SeMet protein) and a = b = 96.9 and c = 301.5 Å (wild-type PA0254). Analysis of the crystallographic data from space group P2_1_ revealed that these crystals show pseudo-merohedral twinning, with a twinning fraction of 0.215. Details of the statistics of data collection are given in Table 1.

**Table 1:** Statistics of data collection and refinement for UbiD2 from *P. aeruginosa*.

	native UbiD2	native UbiD2	Se-Met UbiD2
***Data collection***			
Space group	*P*2_1_	*P*3_2_21	*P*3_2_21
*Cell dimensions*			
a,b,c (Å)	104.3, 55.8, 198.4	96.9, 96.9, 301.5	96.8, 96.8, 301.7
α, β, γ (°)	90.0, 90.9, 90.0	90.0, 90.0, 120.0	90.0, 90.0, 120.0
Wavelength (Å)	0.9763	0.9762	0.9792
Resolution range (Å)	49.60–2.30 (2.42–2.30)	50.3–1.95 (2.06–1.95)	29.75–2.71 (2.86–2.71)
No of observed reflections	314867 (44066)	419182 (62005)	609797 (85905)
No of unique reflections	101115 (14851)	120078 (17286)	45222 (6413)
R^†^ *_merge_*	0.059 (0.244)	0.085 (0.792)	0.077 (0.241)
((I)/s(I))	13.9 (4.1)	7.9 (1.5)	25.1 (10.1)
Completeness (%)	98.9 (99.6)	99.8 (99.6)	99.3 (98.0)
Redundancy	3.1 (3.0)	3.5 (3.6)	13.5 (13.4)
Wilson B-factor (Å^2^)	31.9	28.4	53.8
Twinning fraction	0.215 (h,-k,-l)$		
***Refinement***			
Resolution range (Å)	49.2–2.30	50.3–1.95	
R^‡^_work_/R^§^_free_ (%)	16.4/21.9	19.9/24.8	
No of molecules in a.u.	4	3	
No of atoms	15562	12473	
*Average B-factor (Å^2^)*			
Protein atoms	26.4	38.7	
Water molecules	20.1	40.3	
Sulfate ion	64.1	n/a	
Mg^2+^	n/a	33.3	
*R.m.s. deviations*			
Bond lengths (Å)	0.007	0.008	
Bond angles (°)	1.10	1.24	
*Ramachandran plot*			
favored (%)	97.3	96.0	
allowed (%)	2.6	3.8	
outliers (%)	0.1	0.2	

Values in parenthesis are for the highest resolution shell.

R^†^ *_merge_* = ∑*_hkl_* ∑*_i_* |*I_i_*(hkl)–〈*I*(hkl)〉|/∑*_hkl_* ∑*_i_I_i_*(hkl), where *I_i_*(hkl) is the intensity of an individual reflection and 〈*I*(hkl)〉 is the average intensity for multiple measurements of that reflection. R^‡^ *_work_* = ∑*_hkl_*||*F_o_*_bs_|–||*F_calc_*||/∑*_hkl_* |*F_o_*_bs_|, where F_obs_ and F_calc_ are the observed and calculated structure-factor amplitudes, respectively, for 95% of the reflection data used in the refinement. R^§^ *_free_* = ∑*_hkl_*||*F_o_*_bs_|–||*F_calc_*||/∑*_hkl_* |*F_o_*_bs_| for 5% of the reflection excluded from the refinement.

$ The twinning operator is shown in parenthesis.

### Structure Determination

The structure of SeMet–substituted PA0254 was solved using the MR-SAD protocol of the *Auto-Rickshaw* pipeline [18]. The model used to enhance the phasing comprised a fragment of the A-chain (residues 7–320) of *E. coli* UbiD (PDB code 2IDB), modified in CHAINSAW [19]. The resulting Auto-Rickshaw model of SeMet-PA0254 was further refined using REFMAC5 [20] and manually corrected in COOT [21].

The structure of wild-type PA0254 was solved by molecular replacement using MOLREP [22]. The coordinates of the partially refined model of SeMet-substituted PA0254 without the C-terminal α-helix (residues 469–496) were used as a search model. Initially the structure of the wild-type enzyme was determined and refined in space group *P*2_1_ to 2.3 Å resolution. The molecular replacement gave a clear solution of four subunits in the asymmetric unit and the model was refined using the program PHENIX [23]. Rounds of crystallographic refinement, including TLS and twin refinement, were interspersed with manual model building using COOT [21]. At this stage, a data set to higher resolution from crystals of the trigonal space group (*P*3_2_21) became available. The structure of PA0254 in this crystal form was refined using the same protocol as for the model in space group *P*2_1_, except that twin refinement was excluded as the crystal did not show any signs of twinning. Structure validation was carried out using MolProbity [24]. Details of the refinement statistics and the final models are given in Table 1.

Sequence alignments were performed with ClustalW2 [25] and formatted using ESPript [26]. Figures were prepared using Pymol [27]. The crystallographic data were deposited with the Protein Data Bank with accession codes 4IWS and 4IP2.

## Results and Discussion

### The UbiD Sequence Family

**Figure 2:**
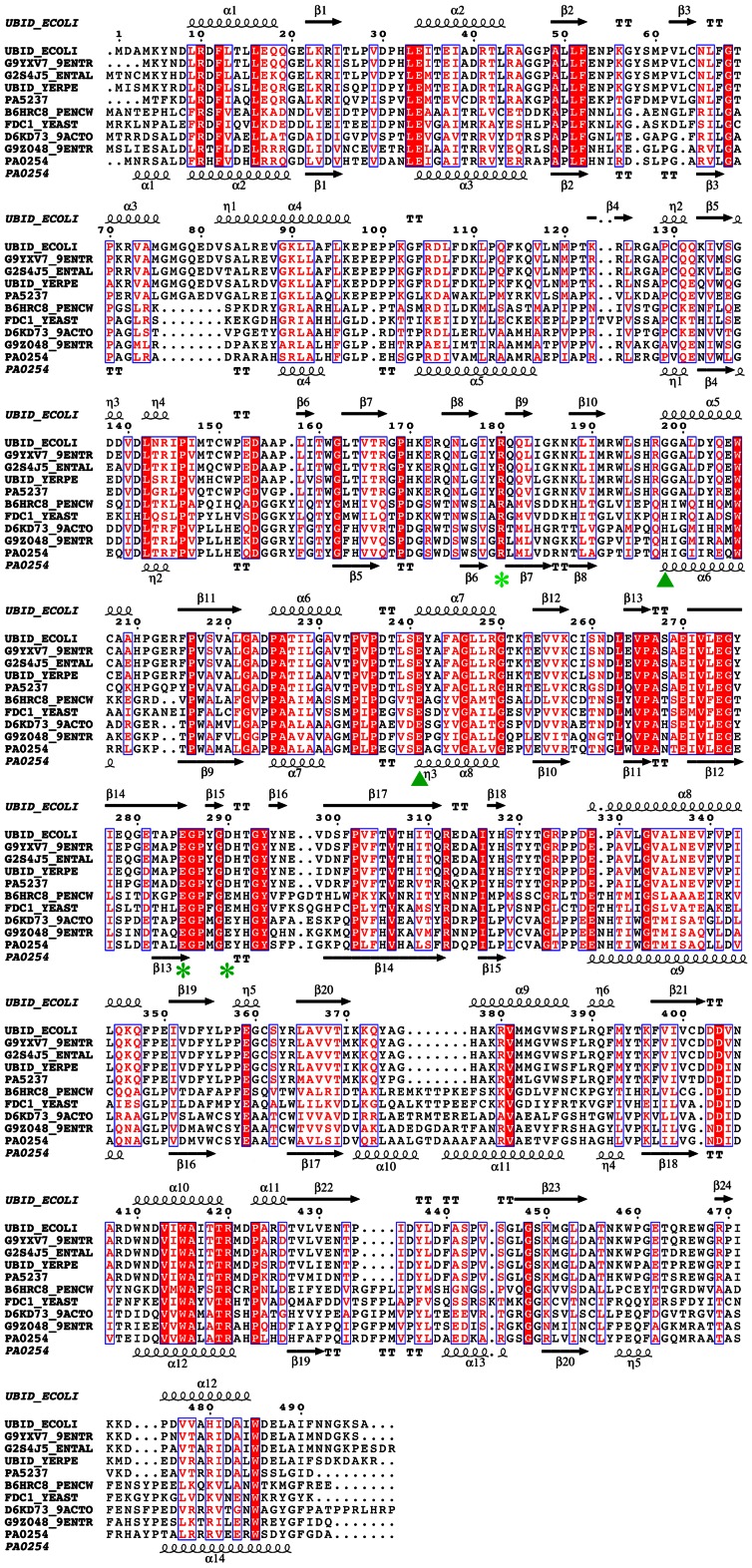
Sequence alignment of UbiD-like proteins. The top five sequences represent *bona fide* UbiD type proteins, and the bottom five sequences are from the subclass of PA0254-like enzymes. Conserved residues are white characters in red boxes; similar residues are shown as red characters in blue frames. Secondary structure elements of UbiD from *E. coli* (PDB code 2IDB) are depicted above the sequence alignment and those of PA0254 from *P. aeruginosa* at the bottom of the alignment. α and 3_10_ helices are displayed as squiggles, β-strands rendered as arrows, β turns by TT and α turns by TTT. Selected sequences are from top to bottom: *bona fide* UbiD from *E. coli*, *Yokenella regensburgei*, *Enterobacter asburiae*, *Yersinia pestis*, *P. aeruginosa* PAO1 (PA5237) and PA0254-like from *Penicillium chrysogenum, Saccharomyces cerevisiae*, *Streptomyces* sp. e14, *Yokenella regensburgei* and *P. aeruginosa* PAO1 (PA0254).The metal ligands and the putative active site residues in UbiD2 are marked by green triangles and asterisks, respectively.

UbiD-like sequences are found in the genomes of most microorganisms, reflecting the ubiquity of the ubiquinone cofactor and the biosynthetic pathway. Most of the microbial sequences annotated as UbiD are very similar to each other and cluster in the range of 70–100% amino acid identities. Figure 2 shows a representative alignment of a subset of UbiD sequences. These enzymes, widely distributed in bacterial and fungal species, form the majority of UbiD-like proteins and are denoted as *bona fide* UbiD in the following. The crystal structure of one representative from this subclass, UbiD from *E. coli* (PDB code 2IDB), determined previously by the Northeast Structural Genomics Consortium, revealed a hexameric quaternary assembly of this enzyme. *P. aeruginosa*, as most other species, contains an *ubiD* like gene, PA5237, which encodes a protein with 76% sequence identity to the *E. coli* enzyme. UbiD from *P. aeruginosa* also shows a hexameric quaternary structure as suggested by size exclusion chromatography (Figure S1in File S1).

*P. aeruginosa* however contains another gene, PA0254 that shows less, but significant sequence identity (24%) to *E. coli* and *P. aeruginosa* UbiD suggesting that it belongs to the same sequence- and fold family as *bona fide* UbiD enzymes. Orthologues of PA0254 are found in each sequenced genome of *P. aeruginosa* strains, but not in other members of the *Pseudomonas* genus. The PA0254 orthologue from *P. aeruginosa* P14, HudA (98% sequence identity), has recently been identified as a virulence-attenuation factor in a *Drosophila melanogaster* model of *Pseudomonas* infection [28]. Sequences closely related to PA0254 (>70% identity) are not as common as UbiD sequences, but relatives (30–45% sequence identity) can be found in several bacterial and fungal genomes (Figure 2). These proteins can be grouped within the UbiD sequence family, but are clearly distinct from the *bona fide* UbiD like enzymes (<25% sequence identity) and form their own subclass, denoted UbiD2 in the following (Figure S2 in File S1).

### Structure Determination of PA0254 from P. aeruginosa and Quality of the Model

The structure of PA0254 from *P. aeruginosa* was determined in two different space groups, *P*2_1_ and *P*3_2_21, to 2.3 Å and 1.95 Å resolution, respectively, by a combination of experimental phasing using SeMet-substituted protein and molecular replacement. The protein models were refined to R_work_/R_free_ values of 16.4/21.9% and 19.9/24.8%, respectively, with good stereochemistry (Table 1). The three (*P*3_2_21) or four (*P*2_1_) polypeptide chains in the asymmetric units, corresponding to a solvent content of 48% or 31%, respectively, are well defined in the electron density maps, except for a few residues at the N- and C-termini and several disordered side chains at the protein surface. In addition, Gly220 in chain C (monoclinic crystal form), located in loop region at the protein surface, is not visible in the electron density map and was therefore not modeled. The final model of the asymmetric unit of the monoclinic crystals contains residues 1–494 (chain A), 2–496 (chain B), 3–219 and 221–494 (chain C), 1–493 (chain D), one sulfate ion and 249 water molecules. The refined model of UbiD2 in the trigonal crystal form contains three polypeptide chains (residues -4–494 in chain A, 0–494 in chain B and -7–494 in chain C; residue numbers -7–0 are amino acids from the His_6_-tag that were disordered to various degrees in the crystal), three Mg^2+^ ions and 784 water molecules. The structures of PA0254 in the two crystal forms are very similar and result in r.m.s.d. values upon superimposition of Cα atoms in the range of 0.7–1.2 Å between the various subunits. Due to the higher resolution the structure of PA0254 as observed in the trigonal crystals was the basis for the following analysis.

### Overall Structure of the UbiD Subunit

**Figure 3:**
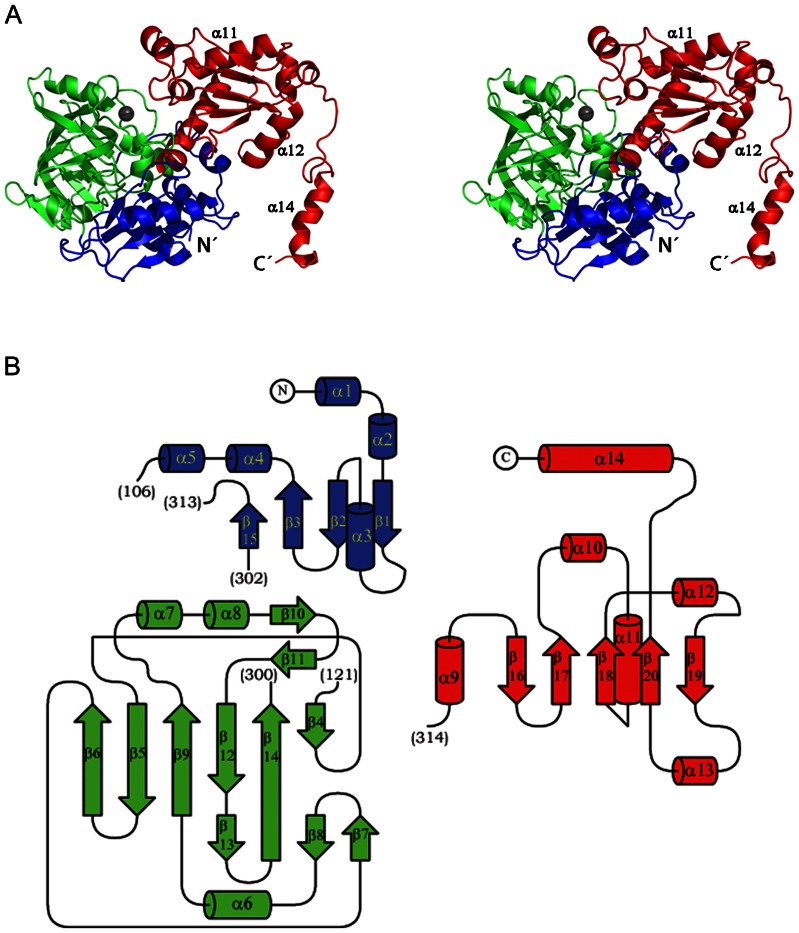
Structure of the *P.aeruginosa* PA0254 subunit. A. Stereo view of the structure of the subunit of PA0254 from *P. aeruginosa*. The three domains are shown in blue (N-terminal domain), green (middle domain) and red (C-terminal domain). The metal site is indicated by a black sphere. N’ and C’ indicate N and C-terminal ends of the polypeptide chain. The helices α11, α12 and α14 involved in dimer formation are indicated. B: Topology diagrams for the three PA0254 domains. The colour-coding is as above.

The PA0254 polypeptide chain folds into a three-domain structure with overall dimensions of the protein core of 43x48x70 Å (Figure 3A). The N-terminal part of the molecule is built up of two domains that pack tightly to each other and form one lobe of the bi-lobal protein. The N-terminal α/β domain (residues 1–106 and 301–313) is built up of a four-stranded, mixed β-sheet, flanked by an α-helix on one side (Figure 3B). After strand β3 the polypeptide chain forms the middle domain (residues 107–300) before completing the β-sheet in the N-terminal domain with the fourth β-strand (β15, residues 301–306). The middle domain consists predominantly of β-structural elements. The core of the domain is formed by a seven-stranded anti-parallel β-sheet that forms a split β-barrel. At one end the barrel is closed off by a capping helix, α6.

The C-terminal domain (residues 314–496) starts with helix α9 that links the middle domain with the C-terminal lobe of the UbiD2 subunit. The core of the domain is a central five-stranded mixed β-sheet (strand order 12354) flanked by two and one helices, respectively, on each side (Figure 3B). The C-terminal residues 462–496 including the C-terminal α-helix (α14) protrude from the core of the molecule (Figure 3A) and are involved in dimer formation (see below).

**Figure 4:**
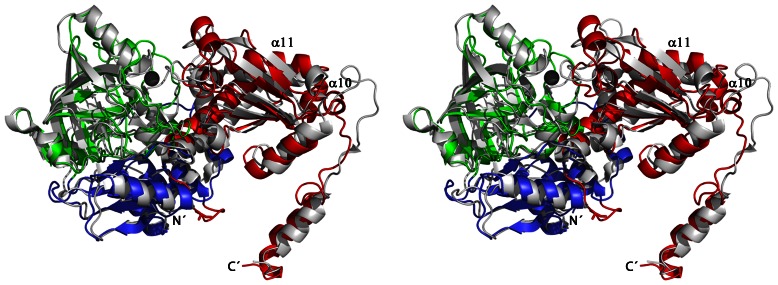
Superimposition of the PA0254 subunit from *P.aeruginosa* (colour-coding as in **figure 3**) with the corresponding subunit of UbiD from *E. coli* (PDB code 2IDB, shown in grey) in stereo. Helices α10 and α11 are labeled, the bound metal ion in PA0254 is depicted as a black sphere.

The overall fold of the *P. aeruginosa* PA0254 subunit is similar to the structure of UbiD from *E. coli* (PDB entry 2IDB), with a DALI [29] Z-score of 40. Superimposition of the subunits results in a r.m.s.d. value of 2.0 Å for 411 aligned Cα atoms. Structural differences are mainly confined to changes in size and/or conformation for several loop regions and a few additional secondary structural elements in the *P. aeruginosa* enzyme, for instance helix α10 after strand β17 and helix α13 preceding strand β20 (Figures 2 and 4).

### Metal Binding Site in PA0254

**Figure 5:**
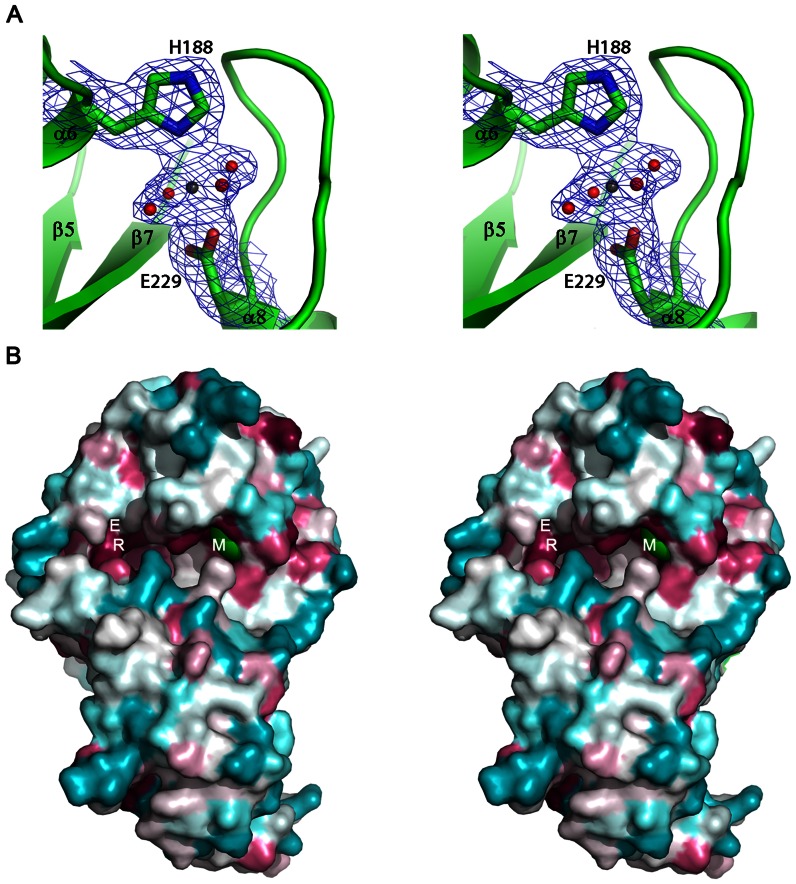
Putative active site of PA0254 from *P.aeruginosa*. A. View of the metal binding site in PA0254. The unbiased, initial 2Fo-Fc electron density map for PA0254 crystallized in space group P3_2_21, obtained after molecular replacement using the metal-free protein model from the P2_1_ crystal form, is contoured at 1.0 σ. The refined model of the metal site is overlaid. His188 and Glu229 are shown as sticks, the bound metal ion is depicted as a black sphere, red spheres represent water molecules. B. Surface representation of the putative active site cleft at the domain interface in PA0254. Residues are colour-coded according to sequence conservation in the UbiD2 family, from the most conserved residues (purple) to the least conserved residues (cyan). Arg170 and Glu273 are indicated by R and E, respectively. The bound magnesium ion (M) is shown as a green sphere. The figure was prepared with the program Consurf [32].

In all three subunits in the asymmetric unit of the trigonal crystal form of PA0254 the initial electron density maps showed clear difference electron density for a metal center (Figure 5A) close to an open cleft at the domain interface. The metal ion is bound in octahedral coordination geometry to the Nδ1 atom of the side chain of His188 and one of the carboxyl oxygen atoms of the side chain of Glu229. Four water molecules complete the ligand sphere. One of these water molecules is in hydrogen bonding distance to the Oγ atom of residue Ser165, making this amino acid a second-sphere ligand to the metal center. The metal-ligand distances are all in the range of 2.0–2.3 Å. The nature of the bound metal ion cannot be determined unambiguously, but based on the ligand geometry, B-factor and electron density it was assigned as a Mg^2+^ ion. The B-factors of the bound Mg^2+^ ions (33 Å^2^) are very similar to those of surround protein residues (30–35 Å^2^). This assignment would also agree with the presence of 0.2 M MgCl_2_ in the crystallization buffer. Noteworthy is also the absence of a bound metal ion in the P2_1_ crystal form, which was obtained without the addition of MgCl_2_ to the crystallization liquor. The residues binding the metal ion are conserved in the PA0254 subclass (Figure 2), suggesting that this metal binding site is present in other members of the UbiD2 subfamily.

Binding of the metal ion induces only localized, moderate changes in conformation in the vicinity of the metal ion. The protein backbone of the loop comprising residues 218–228 shifts its position, with the largest displacement for Pro222 (3.5 Å). The side chains of His188 and Glu229 move slightly (<1 Å) to participate in the ligand sphere of the bound metal. A striking difference in conformation is observed for the side chain of Trp163, which rotates around the Cα-Cβ bond to a clearly different rotamer.

Superimposition of the structures of PA0254 and *E. coli* UbiD shows that the residues forming the metal site are not completely conserved. While Glu229 appears invariant in all sequences of putative UbiDs, His188 is replaced by a glycine residue in the sequences of the *bona fide* UbiD type proteins. The second sphere ligand Ser165 in PA0254 is substituted by an asparagine side chain, which potentially could form a coordination bond to a bound metal ion. However there are no indications in the structure of the *E. coli* enzyme for a bound metal ion at this position.

### Putative Active Site Cleft

The metal binding site is located adjacent to a deep cleft that extends across part of the enzyme surface and may represent the putative active site of the enzyme (Figure 5B). This active site cleft is formed at the interface between the middle and C-terminal domains and amino acid residues from both domains outline the walls of this pocket. The groove has a partly hydrophobic character and is lined by residues Val168, Pro182, Pro185, Ile184, Trp322, Ile326, Leu390, Phe432, Pro433 and Met434 which may be involved in binding of the polyprenyl tail of the substrate 3-nonaprenyl-4-hydroxybenzoate. The metal binding site is located at one end of this cleft. At the opposite end of the groove residues Arg170 and Glu273, which are conserved in the entire UbiD family, are found. These residues, together with another acidic residue, Glu278 (conservatively substituted by an aspartic acid in the *bona fide* UbiD subclass) constitute an evolutionary preserved cluster of hydrophilic residues that might participate in substrate recognition and/or catalysis. So far no *in vitro* biochemical evidence has been obtained demonstrating carboxy-lyase activity for enzymes annotated as 3-polyprenyl-4-hydroxybenzoate decarboxylases of the UbiD family. This is most likely due to the unavailability of the natural substrate, 3-polyprenyl-4-hydroxybenzoate. We could not show decarboxylase activity of PA0254 with the smaller, non-natural substrate analogues vanillic acid, cinnamic acid and ferulic acid. This suggests that presence of a long polyprenyl chain could be required for enzymatic activity, as it presumably facilitates efficient and correct binding of the substrate in the elongated active site pocket.

### Quaternary Structure

**Figure 6:**
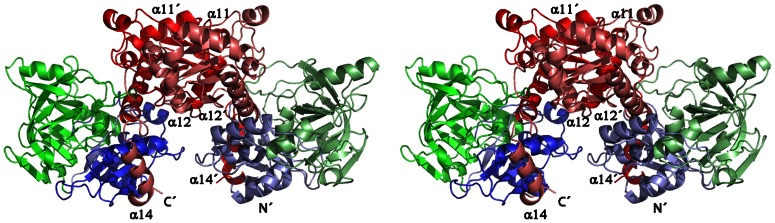
Schematic view of the PA0254 dimer. Chains A and B were colored using the same colour-coding as in figure 3. Helices involved in dimer formation are labeled.

The asymmetric units of the two crystal forms, *P*2_1_ and *P*3_2_21, contain four and three subunits of PA0254, respectively. Manual inspection of crystal packing and analysis using the PISA server [30] showed that PA0254 forms a dimer, with the subunits related by two-fold molecular symmetry (Figure 6). In PA0254 crystals of space group P2_1_, there are two such dimers in the asymmetric unit, whereas in space group P3_2_21 crystals two subunits form a biological dimer within the asymmetric unit and the third subunit forms another dimer with a subunit related by the crystallographic two-fold axis. The calculated molecular mass of His_6_-PA0254 is 57053 Da/subunit and gel filtration data indicate a protein of molecular mass of ∼ 86 kDa, more consistent with a dimeric rather than hexameric assembly of the protein in solution (Figure S1 in File S1).

In total the average interface area of the monomer that becomes buried upon dimerization is ∼ 3500 Å^2^, which accounts for ∼ 17% of the solvent exposed surface area of each subunit. The subunit-subunit interactions in the dimer are mainly mediated through the C-terminal domains, in particular through residues from helix α12 which pack against the β-sheet of the C-terminal domain from the second subunit and *vice versa*. Additional interactions are formed by the C-terminal residues (466–496) including helix α14 that fold over the surface of the N-terminal domain of the second subunit (Figure 6). Many of the interactions at the interface regions are made *via* multiple hydrogen bonds and salt bridges involving ∼ 40 amino acids from each subunit. Several of these contacts engage residues conserved in the sequences of other putative aromatic decarboxylases suggesting that this interface is preserved in the structures from this sequence family. For instance the side chain of Lys393 from one subunit forms multiple hydrogen bonds to carbonyl oxygen atoms of helix α12 of the second subunit and *vice versa* (Figure S3 in File S1). An additional conserved stretch of residues, Gly281-Tyr282, is involved in main chain and side chain hydrogen bonds across the dimer interface. The side chain of Tyr282 forms hydrogen bonds to the side chains of the conserved residues Thr415 and Arg416 of helix α12. A main chain – main chain hydrogen bond is formed between Gly281 of one chain and Ala470 from the second subunit.

The quaternary structure of PA0254 from *P. aeruginosa* differs from that of the *E. coli* enzyme. In the latter, the crystal structure (PDB entry 2IDB) suggests a homo-hexameric assembly with a preserved dimer interface as observed in the *P. aeruginosa* enzyme. The hexamer of *E. coli* UbiD is built up as a trimer of dimers, related by three-fold symmetry (Figure S2 in File S1). Major contributions to the additional interface in the *E. coli* UbiD hexamer are made by helix α9 (residues 377–388, *E. coli* numbering), which packs almost perpendicular against the corresponding helix from another subunit. The loop preceding this helix (residues 374–376) is also part of this interface area and interacts with residues 432–434 from the loop after strand β22 from another subunit.

A superimposition of *P. aeruginosa* PA0254 with the structure of *E. coli* UbiD shows that a corresponding packing into a hexameric assembly is not possible for the *P. aeruginosa* enzyme. This is mainly due to structural differences in the orientation of helix α11 in PA0254 (residues 372–383), corresponding to helix α9 in the *E. coli* enzyme, and the preceding loop 360–371, which also has an insertion of an additional helix α10 in PA0254 (Figure 2). The different orientation of helix α11 relative to the subunit core would result in severe clashes between these helices in the hexamer packing of the *E. coli* enzyme. Thus, a major reason for the observed differences in quaternary structure in the UbiD family appears to be the longer sequence stretch between β17 and α11 in PA0254 including an additional secondary structural element, which results in a different packing of helix α11 in PA0254 compared to the hexameric enzymes.

### Comparison with Related Proteins

Aside from the closest relative, UbiD from *E. coli*, a *DALI* [29] search of the Protein Data Bank using the coordinates of PA0254 identifies a family of flavoproteins as more distant structural relatives with overall Z-scores in the range of 8.8–9.5. These proteins include the NADH:FMN oxidoreductase from *Methylobacillus flagellates* (PDB ID code: 3e4v), the FMN-binding protein from *Methanobacterium thermoautotrophicum* [31] (PDB ID code: 1eje), the putative flavin reductases-like enzyme from *Shewanella baltica* (PDB ID code: 3hmz), the putative flavin reductase from *Shewanella frigidimarin*a (PDB ID code: 3fge), and the nitrilotriacetate monooxygenase component B from *Bacillus cereus* (PDB ID code: 3bpk). These proteins display no significant overall sequence identity to UbiD2 (less than 13%), but nevertheless show structural similarity to the PA0254 middle domain with r.m.s.d values between Cα atoms upon superposition in the range of 2.4–2.9 Å.

In the FMN-binding protein from *M. thermoautotrophicum*, the phosphate group of FMN is anchored to the protein via a metal ion, which in turn is bound to the protein by the side chains of residues His68, Glu105 and Asn142. In UbiD2, two of these residues (His188 and Glu229) are conserved and are involved in binding of the metal ion. However, attempts to demonstrate binding of FMN to PA0254 using spectroscopy and differential scanning fluorimetry were not successful.

### Conclusions

The comparison of UbiD-like sequences suggested that these enzymes can be classified into two different subclasses, denoted *bona fide* UbiD and UbiD2, within this sequence family. The three-dimensional structures of the enzyme subunits are similar in proteins from the two subclasses, but local differences in their primary and tertiary structures have implications for quaternary structure assembly. *Bona fide* UbiD-type proteins form hexamers, whereas UbiD2 enzymes display a dimeric structure. PA0254 contains a Mg^2+^ binding site adjacent to the putative active site cleft. This metal site is also found in a flavin binding protein, which is similar in fold to the middle domain of the UbiD family. In this protein the metal ion is involved in binding of the phosphate group of FMN. However PA0254 appears not to bind FMN and the function of this metal binding site in the UbiD2 family remains enigmatic.

## Supporting Information

Figure S1 Elution profile of PA0254 (UbiD2, top panel) and PA5237 (UbiD1, lower panel) from size exclusion chromatography using a superdex-200 column (GE-Healthcare) calibrated with Ribonuclease-A (13,7 kDa) Chymotrypsinogen-A (25 kDa) Ovalbumin (43 kDa) Albumin (67 kDa) Catalase (232 kDa) and Ferretin (440 kDa). The data for the calibration curve is shown in the insert in the top-panel. The absorbance at 280 nm is displayed as mAU. The elution volume of PA0254 at 75 ml corresponds to a molecular weight of 85 kDa. The peak position of PA5237 at 57 ml indicates a mass of 353 kDa corresponding to a hexameric arrangement.
(PDF)

Figure S2 Phylogenetic tree generated at www.cbrg.ethz.ch/services/PhylogeneticTree based on the sequences of UbiD-like proteins used in the alignment in Figure 2. The members of this sequence family are clustered in two groups, one harboring the hexameric *bona fide* UbiD-like enzymes (to the left) exemplified by UbiD from *E. coli* (PDB code 2IDB). The second group comprises UbiD2-like proteins, represented by dimeric PA0254 described in this paper.
(PDF)

Figure S3 In the dimer interface Lys393 is engaged in interactions with the residues from helix α12 of the second subunit. The ε-amino group of Lys393 potentially forms hydrogen bonds with the carbonyl oxygen atoms of Ala414, Leu413 and Ala417. Hydrogen bonds are displayed as dashed lines with the distances indicated.
(PDF)

File S1 Combined supporting information with Figure S1, Figure S2 and Figure S3.
(PDF)
