# Supplementary material for: Structural Insights into the UbiD Protein Family from the Crystal Structure of PA0254 from Pseudomonas aeruginosa
Source: PLoS One. 2013 May 9;8(5):e63161. doi: 10.1371/journal.pone.0063161 (PMC3650080; doi:10.1371/journal.pone.0063161)
Supplement: Figure S1 — Elution profile of PA0254 (UbiD2, top panel) and PA5237 (UbiD1, lower panel) from size exclusion chromatography using a superdex-200 column (GE-Healthcare) calibrated with Ribonuclease-A (13,7 kDa) Chymotrypsinogen-A (25 kDa) Ovalbumin (43 kDa) Albumin (67 kDa) Catalase (232 kDa) and Ferretin (440 kDa). The data for the calibration curve is shown in the insert in the top-panel. The absorbance at 280 nm is displayed as mAU. The elution volume of PA0254 at 75 ml corresponds to a molecular weight of 85 kDa. The peak position of PA5237 at 57 ml indicates a mass of 353 kDa corresponding to a hexameric arrangement. (PDF) [file pone.0063161.s001.pdf]

Structural insights into the UbiD protein family from the crystal structure of PA0254 from *Pseudomonas aeruginosa*

Agata Jacewicz, Atsushi Izumi, Katharina Brunner, Robert Schnell and Gunter Schneider

Department of Medical Biochemistry and Biophysics, Karolinska Institutet, Stockholm, Sweden.

Supplementary Figure S1.

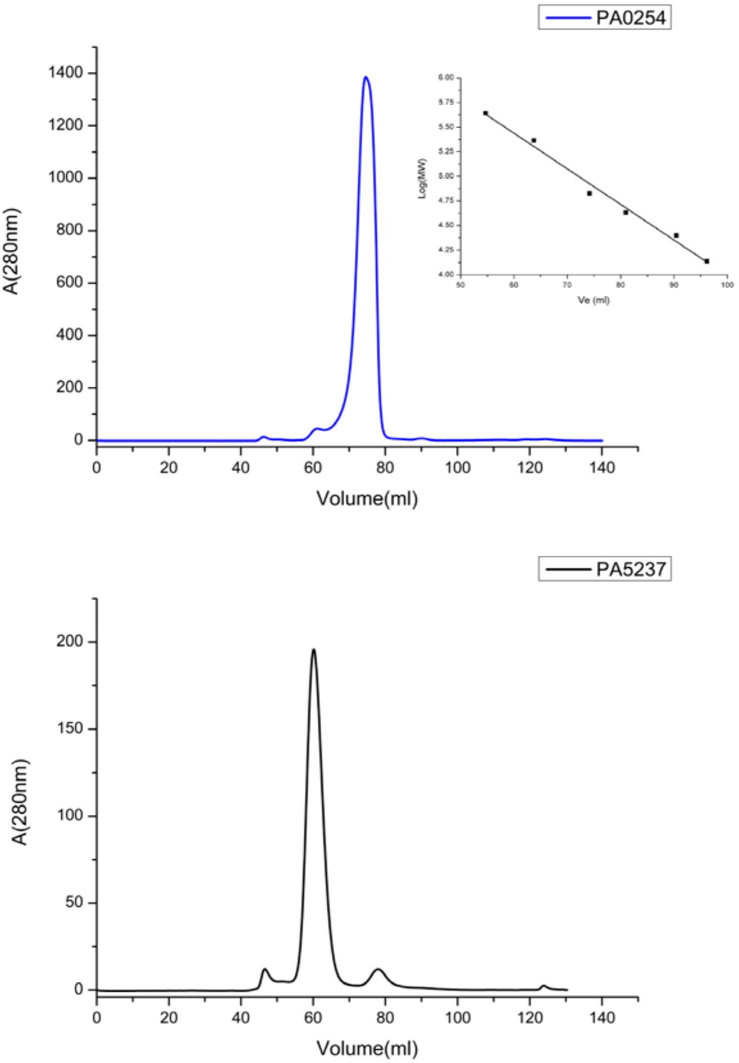

Figure S1. Elution profile of PA0254 (UbiD2, top panel) and PA5237 (UbiD1, lower panel) from size exclusion chromatography using a superdex-200 column (GE-Healthcare) calibrated with Ribonuclease-A (13,7 kDa) Chymotrypsinogen-A (25 kDa) Ovalbumin (43 kDa) Albumin (67 kDa) Catalase (232 kDa) and Ferretin (440 kDa). The data for the calibration curve is shown in the insert in the top-panel. The absorbance at 280 nm is displayed as mAU. The elution volume of PA0254 at 75 ml corresponds to a molecular weight of 85 kDa. The peak position of PA5237 at 57 ml indicates a mass of 353 kDa corresponding to a hexameric arrangement.
