# Supplementary material for: Structural Insights into the UbiD Protein Family from the Crystal Structure of PA0254 from Pseudomonas aeruginosa
Source: PLoS One. 2013 May 9;8(5):e63161. doi: 10.1371/journal.pone.0063161 (PMC3650080; doi:10.1371/journal.pone.0063161)
Supplement: Figure S2 — Phylogenetic tree generated at www.cbrg.ethz.ch/services/PhylogeneticTree based on the sequences of UbiD-like proteins used in the alignment in Figure 2. The members of this sequence family are clustered in two groups, one harboring the hexameric bona fide UbiD-like enzymes (to the left) exemplified by UbiD from E. coli (PDB code 2IDB). The second group comprises UbiD2-like proteins, represented by dimeric PA0254 described in this paper. (PDF) [file pone.0063161.s002.pdf]

**Supplementary Figure S2.**

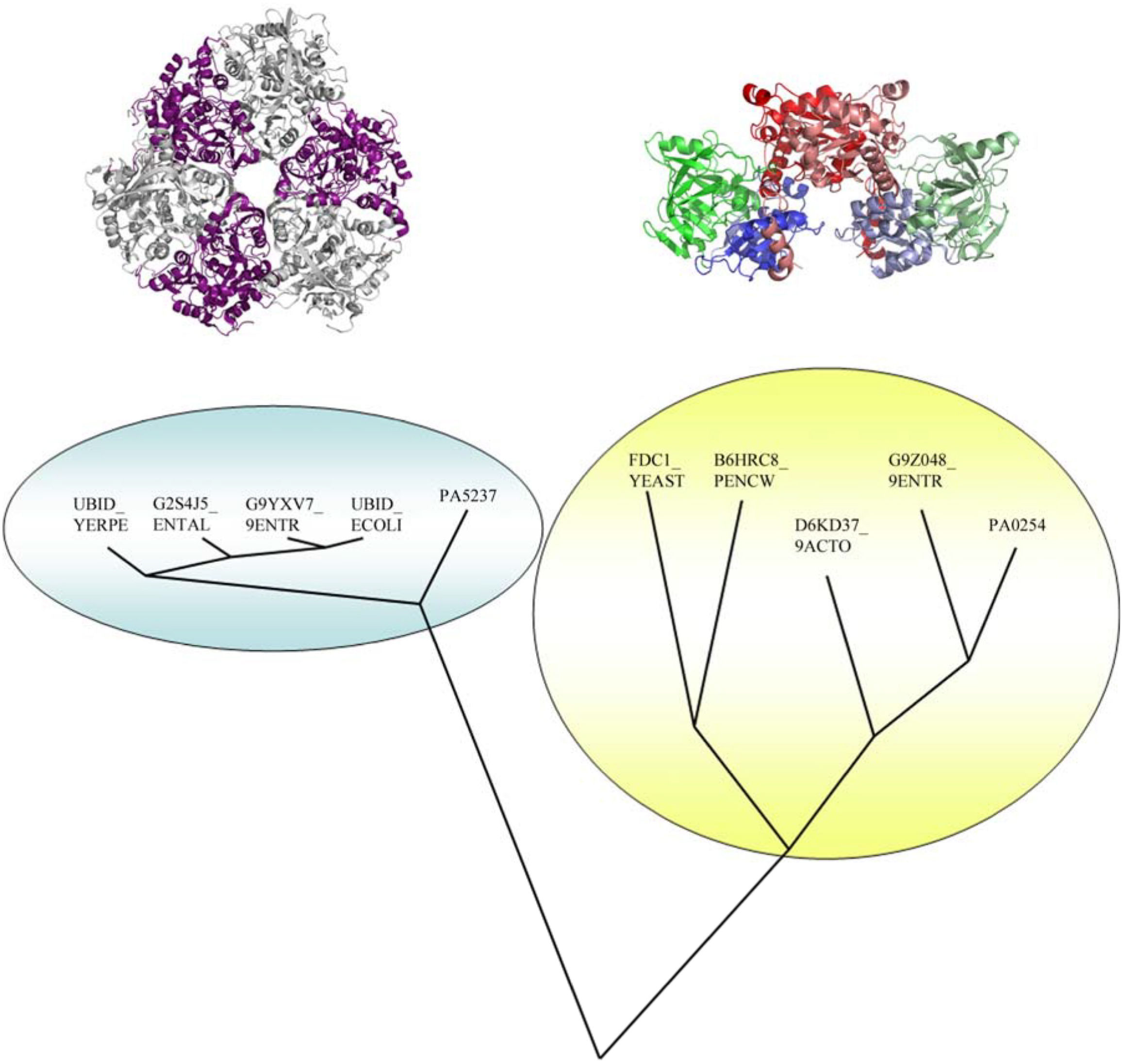


**Figure S2**. Phylogenetic tree generated at [www.cbrg.ethz.ch/services/PhylogeneticTree](www.cbrg.ethz.ch/services/PhylogeneticTree) based on the sequences of UbiD-like proteins used in the alignment in Figure 2. The members of this sequence family are clustered in two groups, one harboring the hexameric *bona fide* UbiD-like enzymes (to the left) exemplified by UbiD from *E. coli* (PDB code 2IDB). The second group comprises UbiD2-like proteins, represented by dimeric PA0254 described in this paper.
