# Supplementary material for: Structural Insights into the UbiD Protein Family from the Crystal Structure of PA0254 from Pseudomonas aeruginosa
Source: PLoS One. 2013 May 9;8(5):e63161. doi: 10.1371/journal.pone.0063161 (PMC3650080; doi:10.1371/journal.pone.0063161)
Supplement: Figure S3 — In the dimer interface Lys393 is engaged in interactions with the residues from helix α12 of the second subunit. The ε-amino group of Lys393 potentially forms hydrogen bonds with the carbonyl oxygen atoms of Ala414, Leu413 and Ala417. Hydrogen bonds are displayed as dashed lines with the distances indicated. (PDF) [file pone.0063161.s003.pdf]

Supplementary Figure S3.

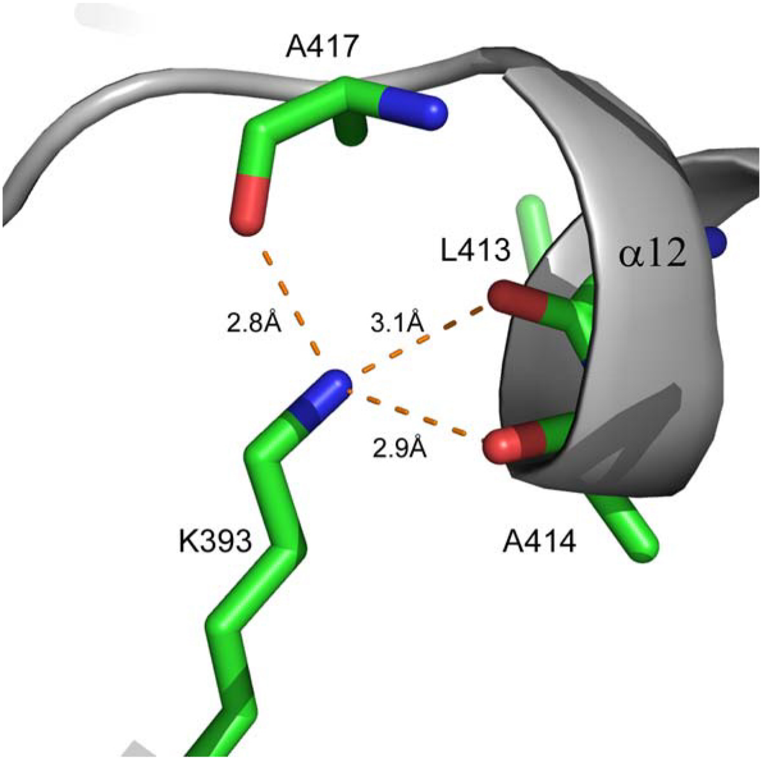

Figure S3. In the dimer interface Lys393 is engaged in interactions with the residues from helix α12 of the second subunit. The ε-amino group of Lys393 potentially forms hydrogen bonds with the carbonyl oxygen atoms of Ala414, Leu413 and Ala417. Hydrogen bonds are displayed as dashed lines with the distances indicated.
