# Supplementary material for: Structural Insights into the UbiD Protein Family from the Crystal Structure of PA0254 from Pseudomonas aeruginosa
Source: PLoS One. 2013 May 9;8(5):e63161. doi: 10.1371/journal.pone.0063161 (PMC3650080; doi:10.1371/journal.pone.0063161)
Supplement: File S1 — Combined supporting information with Figure S1, Figure S2 and Figure S3. (PDF) [file pone.0063161.s004.pdf]

Structural insights into the UbiD protein family from the crystal structure of PA0254 from *Pseudomonas aeruginosa*

Agata Jacewicz, Atsushi Izumi, Katharina Brunner, Robert Schnell and Gunter Schneider

Department of Medical Biochemistry and Biophysics, Karolinska Institutet, Stockholm, Sweden.

Supplementary Figure S1.

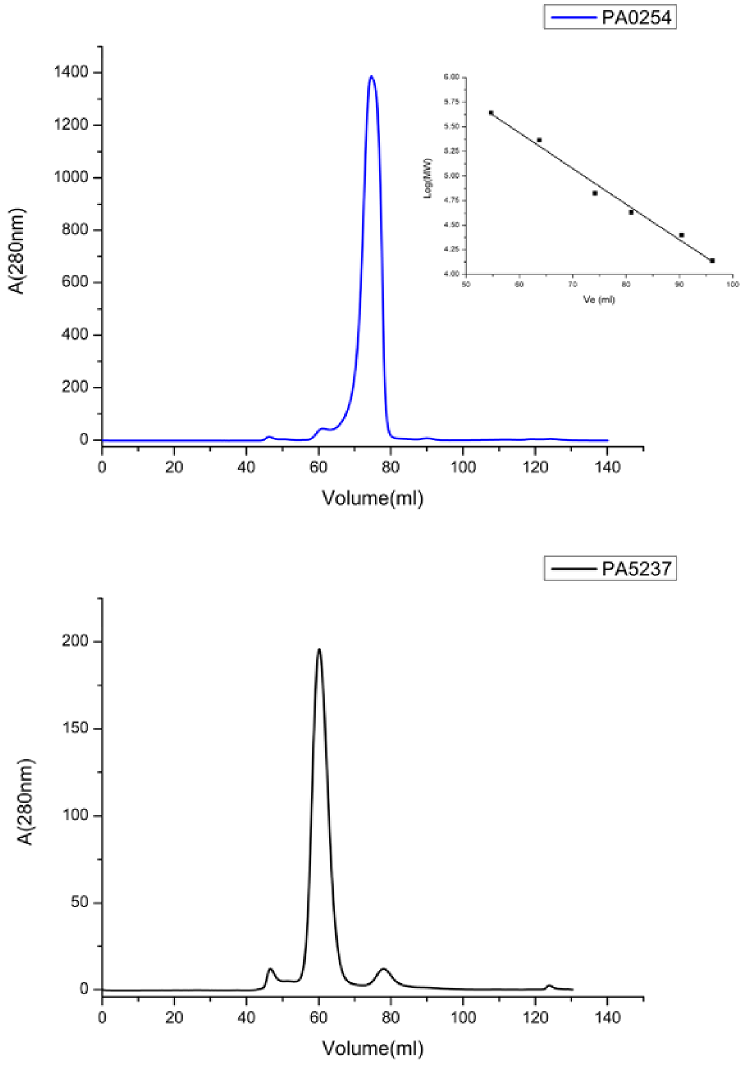

Figure S1. Elution profile of PA0254 (UbiD2, top panel) and PA5237 (UbiD1, lower panel) from size exclusion chromatography using a superdex-200 column (GE-Healthcare) calibrated with Ribonuclease-A (13,7 kDa) Chymotrypsinogen-A (25 kDa) Ovalbumin (43 kDa) Albumin (67 kDa) Catalase (232 kDa) and Ferretin (440 kDa). The data for the calibration curve is shown in the insert in the top-panel. The absorbance at 280 nm is displayed as mAU. The elution volume of PA0254 at 75 ml corresponds to a molecular weight of 85 kDa. The peak position of PA5237 at 57 ml indicates a mass of 353 kDa corresponding to a hexameric arrangement.

Supplementary Figure S2.

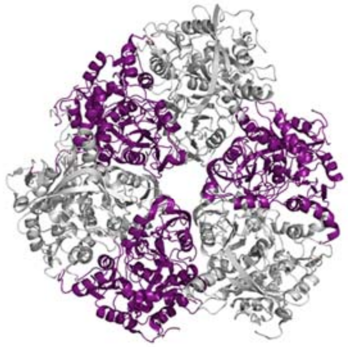

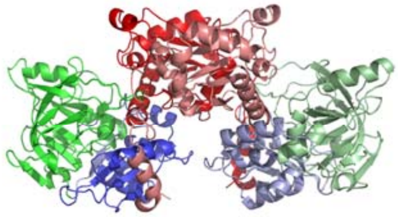

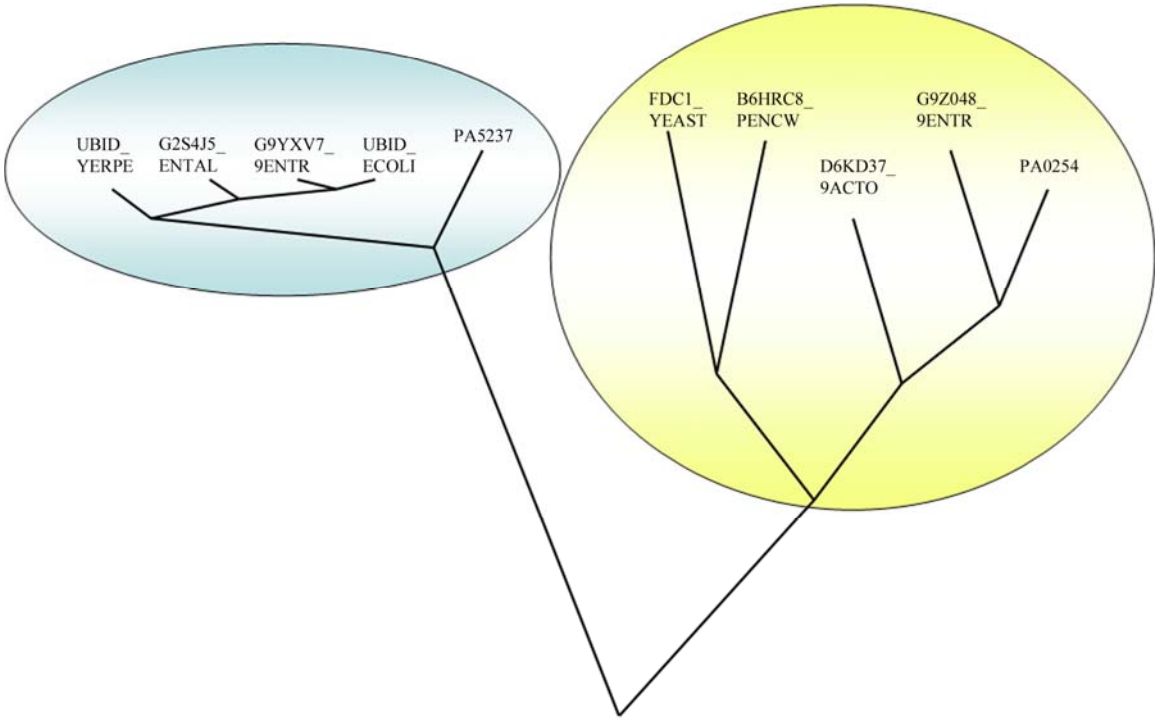

Figure S2. Phylogenetic tree generated at www.cbrg.ethz.ch/services/PhylogeneticTree based on the sequences of UbiD-like proteins used in the alignment in Figure 2. The members of this sequence family are clustered in two groups, one harboring the hexameric *bona fide* UbiD-like enzymes (to the left) exemplified by UbiD from *E. coli* (PDB code 2IDB). The second group comprises UbiD2-like proteins, represented by dimeric PA0254 described in this paper.

Supplementary Figure S3.

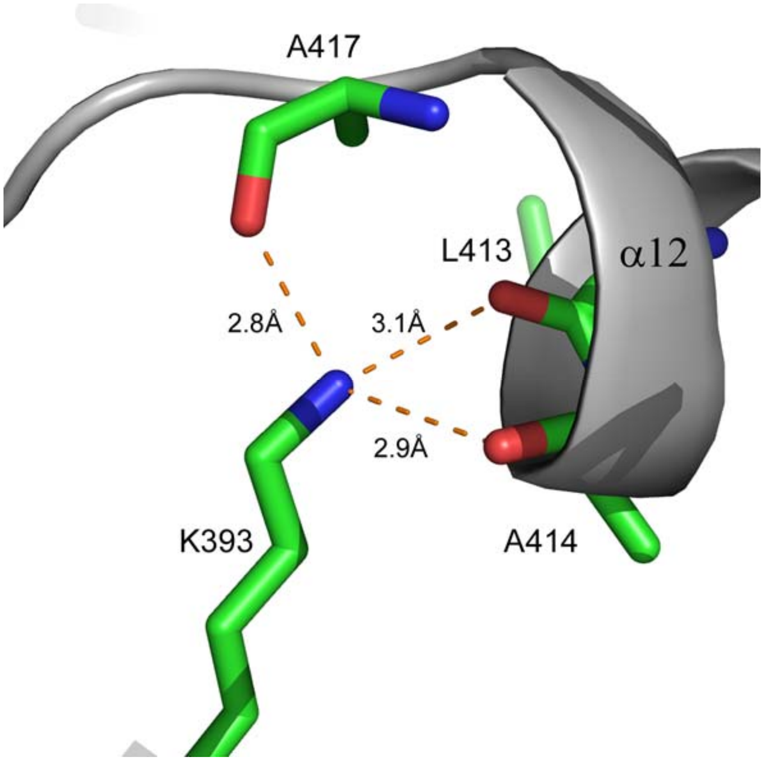

Figure S3. In the dimer interface Lys393 is engaged in interactions with the residues from helix α12 of the second subunit. The ε-amino group of Lys393 potentially forms hydrogen bonds with the carbonyl oxygen atoms of Ala414, Leu413 and Ala417. Hydrogen bonds are displayed as dashed lines with the distances indicated.
